# Cardiac Phosphoproteomics during Remote Ischemic Preconditioning: A Role for the Sarcomeric Z-Disk Proteins

**DOI:** 10.1155/2014/767812

**Published:** 2014-03-30

**Authors:** Safa Abdul-Ghani, Kate J. Heesom, Gianni D. Angelini, M-Saadeh Suleiman

**Affiliations:** ^1^Bristol Heart Institute & School of Clinical Sciences, Faculty of Medicine & Dentistry, University of Bristol, Bristol Royal Infirmary, Level 7, Marlborough Street, Bristol BS2 8HW, UK; ^2^The Proteomics Facility, Medical Sciences Building, University Walk, Bristol BS8 1TD, UK

## Abstract

Remote ischemic preconditioning (RIPC) induced by brief ischemia/reperfusion cycles of remote organ (e.g., limb) is cardioprotective. The myocardial cellular changes during RIPC responsible for this phenomenon are not currently known. The aim of this work was to identify the activation by phosphorylation of cardiac proteins following RIPC. To achieve our aim we used isobaric tandem mass tagging (TMT) and reverse phase nanoliquid chromatography tandem spectrometry using a Linear Trap Quadropole (LTQ) Orbitrap Velos mass spectrometer. Male C57/Bl6 mice were anesthetized by an intraperitoneal injection of Tribromoethanol. A cuff was placed around the hind limb and inflated at 200 mmHg to prevent blood flow as confirmed by Laser Doppler Flowmetry. RIPC was induced by 4 cycles of 5 min of limb ischemia followed by 5 min of reperfusion. Hearts were extracted for phosphoproteomics. We identified approximately 30 phosphoproteins that were differentially expressed in response to RIPC protocol. The levels of several phosphoproteins in the Z-disk of the sarcomere including phospho-myozenin-2 were significantly higher than control. This study describes and validates a novel approach to monitor the changes in the cardiac phosphoproteome following the cardioprotective intervention of RIPC and prior to index ischemia. The increased level of phosphorylated sarcomeric proteins suggests they may have a role in cardiac signaling during RIPC.

## 1. Introduction

**Figure 1:**
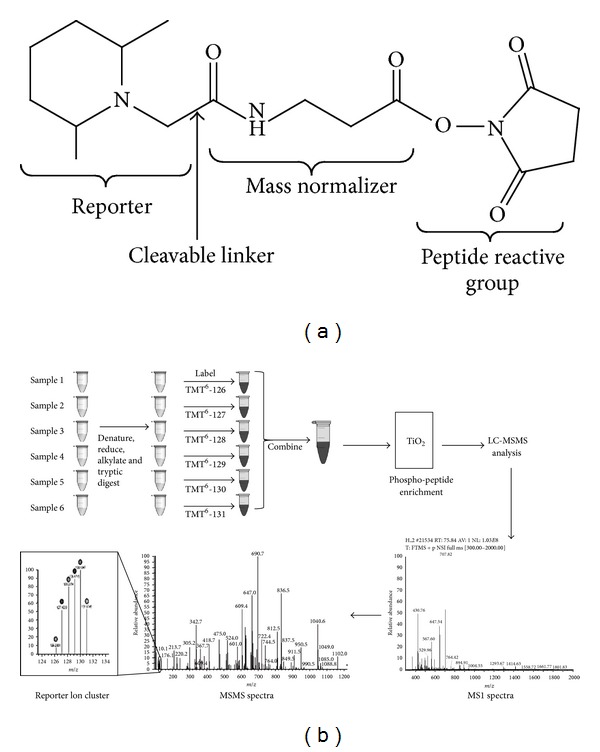
Phospho-TMT workflow. (a) Structure of the TMT Tag. Six tags are available, each with a different reporter group (*m/z* 126, 127, 128, 129, 130, or 131). The mass normaliser region balances out the difference in mass in the reporter groups such that the overall tag mass is constant. The reactive group provides amine-specific labelling. (b) Six samples are digested with trypsin to generate peptides which are then labelled with one of the six TMT tags. The labelling reaction is quenched and the samples are pooled. The pooled sample is passed through a titanium dioxide (TiO_2_) column, phosphopeptides bind to the column while nonphosphorylated peptides pass straight through. The phosphopeptides are then eluted and analysed by LC-MS/MS. Differentially tagged peptides are indistinguishable at the MS1 level since the overall tag mass is constant. Fragmentation of the peptides detected in the MS1 spectra produces secondary MSMS spectra for each peptide, allowing elucidation of the peptide sequence. In addition, the fragmentation process causes cleavage of the linker region within the tag, releasing the reporter groups which appear as a cluster of ions at the low mass end of each MSMS spectra. The relative intensity of the members of this ion cluster shows the relative abundance of that peptide between the six samples under comparison.

Remote ischemic preconditioning (RIPC) is a powerful protective phenomenon in which brief ischemic periods of a remote organ (e.g., arm or leg) confers protection of another organ (e.g., heart) against a sustained ischemia-reperfusion (I/R) insult (reviewed by [1]). Clinical benefits of RIPC have been demonstrated in patients undergoing primary percutaneous coronary intervention [2–6]. The benefits have also been demonstrated in both pediatric [7, 8] and adult open heart surgery [9–14]. Meta-analysis further confirmed the cardioprotection by RIPC in adult cardiac surgery [15] and its potential clinical application is expected to be excellent [16]. However, the mechanism(s) underlying this intervention and how a preconditioning stimulus in a limb confers protection to the patients' heart is essential for maximizing the beneficial effects of RIPC. Essentially the preconditioned organ transmits a signal to the heart which in turn triggers changes in the myocardium that eventually results in protection. Several mechanisms have been proposed that are based on experimental models (reviewed by [1, 17]). For example it has been suggested that the contact between the remote preconditioned organ/tissue and the heart could include humoral [18–20], neural factors [20, 21], and systemic changes. It is suggested that protection by RIPC is triggered by release of metabolites (e.g., adenosine, bradykinin, and opioids) from the remotely preconditioned organ [22–26]. In fact recent work has shown that adenosine A1 receptors are directly involved in RIPC [27]. However, little is known about the changes in target tissue (myocardium) as a result of RIPC and* prior* to I/R. Most studies have largely reported changes in signaling pathways after reperfusion. These include the activation of intracellular kinases such as PKC*ε* [28–30], activation of the reperfusion injury salvage kinase (RISK) pathway and the survivor activating factor enhancement (SAFE) pathway in early reperfusion [31]. Several of these pathways are similar to what happens in ischemic preconditioning. In contrast, little is known about the activation of signaling pathways immediately following RIPC and prior to I/R. It has been recently proposed that phosphorylation of key proteins are essential for RIPC as hypoxic hearts of pediatric patients do not result in further increase in phosphorylation of key signaling proteins and thus were not protected by RIPC [32]. In order to highlight the importance of phosphorylation in RIPC, we used our recently characterized RIPC mouse model [33] to monitor the changes in the activation (phosphorylation) of cardiac proteins following RIPC. Therefore we studied the cardiac phosphoproteome in both RIPC and in control hearts using Tandem Mass Tagging (TMT). TMT is an isobaric mass tagging approach commonly used for quantitative proteomics which allows the comparison of up to six different samples in a single experiment [29]. With this approach, samples are labelled after extraction, making it applicable to the comparison of nonculturable samples unlike the more traditional metabolic labelling approaches such as stable isotope labelling with amino acids in cell culture (SILAC). Labelling is performed at the peptide level using a set of six amine-specific isobaric tags which covalently attach to the peptide N-terminus (and the amino group of lysine residues), thereby labelling all peptides in a given sample. Each isobaric tag consists of a unique reporter group (of* m/z* 126, 127, 128, 129, 130 or 131) and a mass normaliser which ensures that the overall mass of each tag is the same (see Figure 1 in Methods). Thus differentially labelled peptides are identical in mass (and therefore indistinguishable) at the Mass spectrometry MS1 level. However, during the liquid chromatography followed by tandem mass spectrometry (LC-MS/MS) analysis, fragmentation of each peptide not only generates an MS/MS spectra from which the amino acid sequence of the peptide is determined, but also results in cleavage and release of the reporter groups which appear as a cluster of ions at the low mass end of each MSMS spectra. The relative intensity of each reporter ion in that cluster is then used to give a measure of the relative abundance of that peptide between the six samples under comparison. We have combined this approach with the use of titanium dioxide (TiO_2_) for phosphopeptide enrichment, thus allowing us to compare the relative levels of individual phosphopeptides between control and intervention (RIPC) samples.

## 2. Methods

### 2.1. Animals

Male C57/BL6 wild-type mice (25–28 weeks old, 26–33 g) were used for all experiments. All animals were purchased from B&K Universal. Animals were kept at the University of Bristol Veterinary School until used. Treatment of animals and all procedures were in accordance with Home office guidance (Scientific Procedures) Act of 1986.

### 2.2. RIPC Model

The characteristics of RIPC mouse model have been published elsewhere [33]. In brief, mice were anesthetized by an intraperitoneal injection (0.020 mL/g weight) of 2.5% tribromoethanol (Avertin) (Sigma-Aldrich, UK), allowed five minutes to become fully anesthetized (evidenced by lack of response to toe or tail pinch). A specially designed small pressure cuff (1.6 × 9 cm) (Hokanson, Inc.) was placed around the hind limb at the inguinal level. Blood flow in the hind limb was monitored using laser Doppler flowmetry (moorLDI2 imager) and was also confirmed by the change in the leg skin color. Body temperature was maintained at around 37°C using a heating pad.

### 2.3. RIPC Protocol

RIPC was induced by 4 cycles of 5 min of limb ischemia at 200 mmHg followed by 5 min of reperfusion (*n* = 6). Control group (*n* = 6) had a deflated cuff placed on the lower limb. At the end of the procedure (50 min), the animals were immediately terminated by cervical dislocation and the heart extracted and ventricular tissue snap frozen in liquid nitrogen and stored at −80°C. The maximum number of samples that can be analysed in a single tandem mass tagging experiment is six. Therefore, we carried out two separate runs to analyze the 12 samples. Each run (Series) involving six samples, included three control and three RIPC samples. Fold change between RIPC and control were calculated separately for each series.

### 2.4. Protein Extraction and Quantification

Each ventricular tissue was weighed and added to lysis buffer containing (1% Noidet P-40/IGEPAL CA-630, 0.5% sodium deoxycholate, 0.1% SDS in 1x PBS) and one tablet of each proteases and phosphatases inhibitor cocktails at a ratio of 10 *μ*L per mg of tissue. The mixture was kept on ice. The biopsies were then homogenized at high speed for 12 sec (twice) using Minilys tissue homogenizer (Bertin technologies, France) which involved the use of bead beating technology. The homogenate was left 30 minutes shaking on ice, at 4°C and then centrifuged at 10,000 g for 10 minutes at 4°C and the supernatant was collected. Protein concentration in the supernatant was determined with the total protein kit, Micro Lowry (Sigma, UK). Bovine serum albumin (BSA) at a stock concentration of 5 mg/mL was used to obtain a standard curve. For each sample, 400 *μ*L of Lowry reagent was added and incubated at room temperature for 30 min, followed by adding 200 *μ*L of Folin-Ciocalteu reagent incubated for 30 min. The absorbance was measured at 750 nm using spectrophotometer (Jenway7305, UK). Protein concentration in samples was determined by interpolating the densities from the standard reference curve.

### 2.5. Immunoblotting

Cardiac samples from Series 1 were used for both western blotting and phosphorproteomics (see below). Reducing sample buffer (5x, 4% SDS v/v, 0.1 M Tris-HCl pH 6.8, 10% mercaptoethanol, 25% glycerol) was added to the protein samples and was heated at 95°C for 5 min. In contrast proteins extracted for western blot to measure the phosphorylated phospholamban (PLB) were not boiled as per the manufacturer's protocol (Abcam, see below). 20 *μ*g/mL of total protein were separated on 4–20% gradient gel (Mini-ProteanTGX, Bio-Rad, UK) and revealed using Western blotting with antibodies (diluted 1 : 2000) against P-PLB (Abcam ab15000 (Ser16)), myozenin-2 (Santa Cruz sc-377359) at a dilution of 1 : 1000 and with glyceraldehyde 3-phosphate dehydrogenase (Cell signalling 5174) at a dilution of 1 : 10000. The membranes were incubated with horseradish peroxidase-conjugated secondary antibody at a dilution of 1 : 10000 for 1 h at room temperature. Immunoreactivity was visualized with an ECL reagent (ECL prime western blotting reagent, Amersham, GE Healthcare). They were then exposed to X-ray film. Band intensity was quantified by densitometry using Image J (National Institutes of Health, USA) and the resulting peptides were normalized to GAPDH. Immunoblotting was carried out on samples from the first proteomic run (series 1: 3 control and 3 RIPC).

### 2.6. Phosphoproteomic Analysis

Analysis of protein phosphorylation in ventricular tissues extracted from both RIPC and control groups was performed using Tandem Mass Tags (TMTs) from Thermo Fisher Scientific (University of Bristol Proteomics Facility, School of Medical Sciences). Details of the methods involved are shown in Figure 1. The analysis was performed in all twelve hearts used in this study (6 control and 6 RIPC). However, as we could only run 6 samples at a time (3 RIPC and 3 control) the whole processing involved two runs (2 Series). Moreover, the comparison including statistics could only be performed comparing samples (3 control versus 3 RIPC) within one series.

#### 2.6.1. TMT Labelling and Phosphopeptide Enrichment

Aliquots of 100 *μ*g of six samples per experiment were digested with trypsin (2.5 *μ*g trypsin per 100 *μ*g protein; 37°C, overnight) and labelled with Tandem Mass Tag (TMT) 6Plex reagents according to the manufacturer's protocol (Thermo Fisher Scientific, Loughborough, LE11 5RG, UK). Each sample was labelled with a different isobaric tag, RIPC samples were labelled as TMT126, 127, and 128 and for control samples TMT129, 130, and 131. The labelled samples were then combined, evaporated to dryness, resuspended in buffer (57% acetonitrile v/v, 0.4% TFA v/v, 26% lactic acid v/v), and subjected to phosphopeptide enrichment using titanium dioxide TiO_2_ Phosphopeptide enrichment kit (Pierce).

#### 2.6.2. Nano-LC Mass Spectrometry

Enriched phosphopeptides were then fractionated using a Dionex Ultimate 3000 nanoHPLC system in line with an LTQ-Orbitrap Velos mass spectrometer (Thermo Scientific). In brief, peptides in 1% (vol/vol) formic acid were injected onto an Acclaim PepMap C18 nanotrap column (Dionex). After washing with 0.5% (vol/vol) acetonitrile 0.1% (vol/vol) formic acid peptides were resolved on a 250 mm × 75 *μ*m Acclaim PepMap C18 reverse phase analytical column (Dionex) over a 150 min organic gradient, using 7 gradient segments (1–6% solvent B over 1 min, 6–15% B over 58 min, 15–32% B over 58 min, 32–40% B over 3 min, 40–90% B over 1 min, held at 90% B for 6 min, and then reduced to 1% B over 1 min) with a flow rate of 300 nl min^−1^. Solvent A was 0.1% formic acid and Solvent B was aqueous 80% acetonitrile in 0.1% formic acid. Peptides were ionized by nanoelectrospray ionization at 2.0 kV using a stainless steel emitter with an internal diameter of 30 *μ*m (Thermo Scientific) and a capillary temperature of 250°C. Tandem Mass Spectra were acquired using an LTQ- Orbitrap Velos mass spectrometer controlled by Xcalibur 2.1 software (Thermo Scientific) and operated in data-dependent acquisition mode. The Orbitrap was set to analyse the survey scans at 60,000 resolution (at* m/z* 400) in the mass range* m/z* 300 to 1800 and the top ten multiply charged ions in each duty cycle selected for MS/MS fragmentation using higher-energy collisional dissociation (HCD) with normalized collision energy of 45%, activation time of 0.1 ms and at a resolution of 7500 within the Orbitrap. Charge state filtering, where unassigned precursor ions were not selected for fragmentation, and dynamic exclusion (repeat count, 1; repeat duration, 30 s; exclusion list size, 500) were used.

The raw data files were processed and quantified using Proteome Discoverer software v1.2 (Thermo Scientific) and searched against the UniProt Human database (122604 entries) using the SEQUEST (Ver. 28 Rev. 13) algorithm. Peptide precursor mass tolerance was set at 10 ppm, and MS/MS tolerance was set at 0.8 Da. Search criteria included oxidation of methionine (+15.9949) and phosphorylation at serine, threonine, and tyrosine (+79.966) as variable modifications and carbamidomethylation of cysteine (+57.0214) and the addition of the TMT 6Plex mass tag (+229.163) to peptide N-termini and lysine as fixed modifications. Searches were performed with full tryptic digestion and a maximum of 1 missed cleavage was allowed. The reverse database search option was enabled and all peptide data was filtered to satisfy false discovery rate (FDR) of 5%. The Proteome Discoverer software generates a reverse “decoy” database from the same protein database and any peptides passing the initial filtering parameters that were derived from this decoy database are defined as false positive identifications. The minimum cross-correlation factor (Xcorr) filter was readjusted for each individual charge state separately to optimally meet the predetermined target FDR of 5% based on the number of random false positive matches from the reverse decoy database. Thus each data set has its own passing parameters. Quantitation was done using a peak integration window tolerance setting of 0.0075 Da with the integration method set as the most confident centroid.

### 2.7. Statistical Analysis

Statistical analyses were performed using Statveiw for Windows (SAS Institute Inc.). Differences between control and RIPC groups were analyzed using unpaired* t*-test. Since there were two phosphoproteomic runs with each involving six different samples (3 control and 3 RIPC), the comparison was only made between the two groups for each run.

## 3. Results

### 3.1. The Effect of RIPC on Cardiac Phospho-Proteins in Series 1

The first phospho-proteomic analysis run was carried out on 6 samples (3 control and 3 RIPC). Out of a total of ~1700 phosphoproteins measured, only 15 phosphoproteins showed significant increase in RIPC compared to control (Table 1). Most of these proteins are signalling molecules localized to the cardiac sarcomeric Z-disk. Interestingly the RIPC-induced changes in phosphoproteins were similar whether the absolute values were used or when expressed (normalised) per phosphorylated-GAPDH. The latter was detected in all samples.

### 3.2. Validation of Phosphoprotein Levels in Series 1 Using Western Blotting

Whether the changes in phosphoproteins is a real effect that was validated using western blotting on the same samples that were used for phosphoproteomics. We selected phosphorylated phospholamban to validate phosphoproteomics analysis by western blotting for a number of reasons. First, we detected this protein in the phosphoproteome in all samples. This protein showed significant variation which raised concern as to the phosphoproteomic analysis. Finally, we have the antibody for this protein (phosphorylated at Ser16) which we use routinely in our studies and therefore are very confident using it (Figure 3).

**Figure 2:**
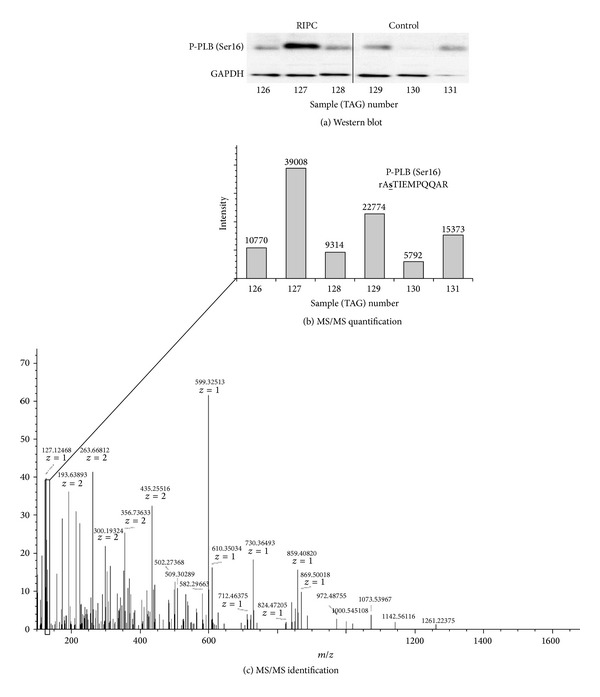
Quantification of phosphorylated phospholamban (p-PLB) in the same samples measured using Western blotting or TMT tandem mass tagging. Protein expression level for cardiac p-PLB (GAPDH in lower panel) in RIPC (126, 127, and 128) and control (129, 130, and 131) samples are shown in (a). Raw data for identification (c) and quantification (b) of the phosphopeptide RASTIEMPQQAR from PLB using TMT isobaric mass tagging are shown in (b) & (c). The reporter ion cluster from the six different mass tags are shown boxed in panel (c) and expanded in bar chart form in panel (b) for both RIPC (numbers: 126, 127, and 128) and control (129, 130, and 131) samples. The peptide sequence showing the phosphorylated amino acid (Ser 16, underlined) is also shown in panel (b). The samples used in these measurements were taken from series 1.

**Figure 3:**
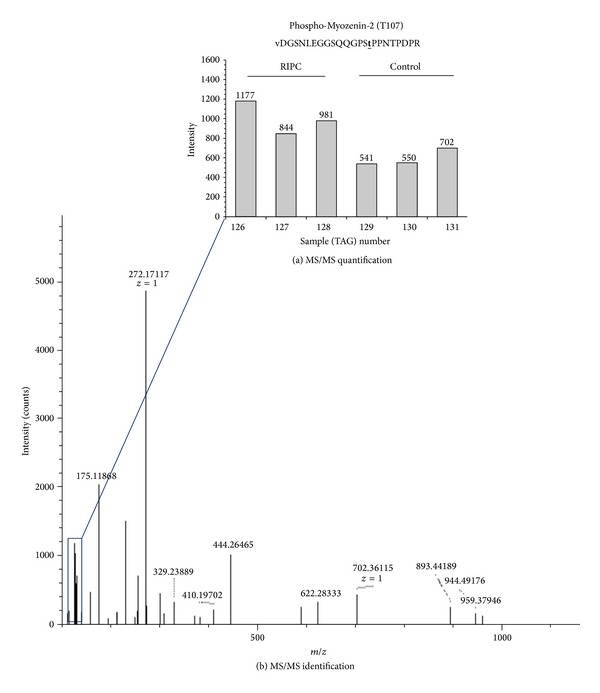
Phosphopeptide identification and quantification for phospho-myozenin-2 from RIPC and control samples using Tandem Mass Tagging. P-myozenin-2 peptide phosphorylated at threonine 107 is shown in panel (a). The mass peaks generated by different mass tags (reporter ions) for different samples are magnified and shown in bar chart form in panel (a) for both RIPC (numbers: 126, 127, and 128) and control (129, 130, and 131) samples. The (MS/MS) spectra for the peptide used for protein identification and the reporter ions (boxed) are shown in (b). The samples used in these measurements were taken from Series 1.

The absolute levels of p-PLB measured for individual samples using TMT isobaric mass tagging were compared to levels measured using Western blot for p-PLB and corrected for GAPDH. As shown in Figure 2 the levels of p-PLB in different samples followed a similar pattern whether using phosphoproteomics or Western blotting.

### 3.3. The Effect of RIPC on Cardiac Phosphoproteins in Series 2

The second phosphoproteomic analysis run was carried out on an extra, different 6 samples (3 control and 3 RIPC). The analysis of these new samples detected ~2300 phosphoproteins. However, only 14 phosphoproteins showed significant differences between RIPC and control (Table 2). Most of these proteins are also signalling molecules localized to the cardiac sarcomeric Z-disk. The protein that showed the highest change in the level of phosphorylation in both series was myozenin-2 (also known as Calsarcin 1). This is a novel family of sarcomeric calcineurin-binding proteins.

### 3.4. The Effect of RIPC on Myozenin-2 (Calsarcin1) Phosphorylation

**Figure 4:**
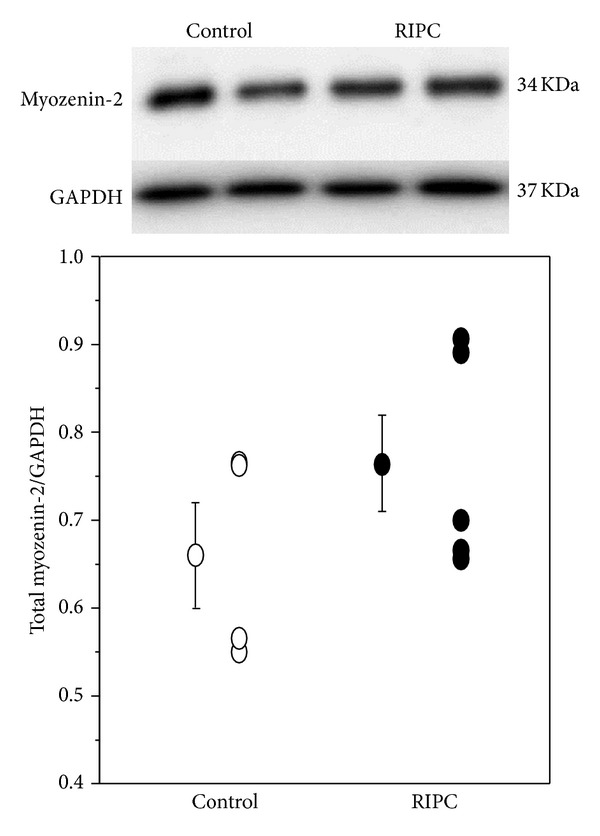
Total myozenin-2 in ventricular tissues extracted from RIPC (*n* = 5) and control (*n* = 4) samples. Upper panel is a representative blot showing myozenin-2 and GAPDH. The graph shows individual myozenin-2 levels (normalized to GAPDH) as well as the mean ± SEM for each intervention. There was no statistical difference between the two groups.

RIPC significantly increased myozenin-2 phosphorylation compared to the control group in both runs (Tables 1 and 2). The following phosphorylation sites were detected; threonine 107, serine 106, and serine 101 (Table 3). Unlike phosphorylated myozenin-2, the total myozenin-2 measured using western blotting did not show significant changes between RIPC and control (Figure 4). There are no antibodies available for phosphorylated myozenin-2 and therefore did not perform western blot for this protein.

## 4. Discussion

The mechanism by which RIPC protects the myocardium against ischemia/reperfusion (I/R) injury is not clear; however, some proposed pathways suggest the release of substances from the remote organ which activate a complex intracellular signaling cascade in myocardium. It is proposed that these signalling pathways act either via the activation of the potassium-dependent ATP (K_ATP_) channel [34] and/or by inhibiting the opening of the mitochondrial permeability transient pore to induce cardioprotection [35]. The involvement of these pathways has been demonstrated during reperfusion with little work describing protein activation at the end of RIPC and before I/R. Therefore we performed a phosphoproteomic study to identify proteins that are activated immediately after RIPC.

Cardiac proteomic studies provide insight into changes in total protein expression during cardiac disease and for the identification and localization of posttranslational modifications with the hope that this technique will also help to identify markers of cardiac disease as well as novel therapeutic targets [36–41]. Two-dimensional gel electrophoresis has been used to visualize the proteome profile in the border zone of the early stage postinfarct [42] and proteins that were differentially expressed in the ischemically reperfused heart compared with the control and ischemically preconditioned rat hearts [43]. Proteomic blood study during RIPC study using 2D-gel suggested either neurogenic pathway or humoral factor less than 8 kDa as possible mediators [44].

A recent study by Hepponstall et al. (2012) examined the global proteomic changes that occur during RIPC in plasma. They identified six proteins that changed in response to RIPC using 2D DIGE analysis, while 48 proteins were found to be differentially regulated using LC-MS [45]. These changes were cumulative with each episode of RIPC and the proteins identified have a range of cellular functions including immune response, homeostasis, cell adhesion, and lipid transport. Recent years have witnessed the introduction of gel-free proteomic approaches. These include isotope-coded affinity tagging (ICAT), SILAC, and isobaric tag for relative and absolute quantification (iTRAQ) [46]. These LCMS-based approaches provide considerably more information than the more classic 2D gel-based approaches.

In our study we used Tandem Mass Tagging, (TMT) combined with phosphopeptide enrichment to determine the changes in the cardiac phosphoproteome during the cardioprotective intervention, RIPC. The technique was also confirmed and validated using western blotting. Several of the phosphoproteins identified were associated with Z-disk sarcomere.

Our phosphoproteomic analysis showed that RIPC is associated with an increase of phosphorylation of several proteins most of which are found in the Z-disk of the sarcomere including: myozenin-2, Obscurin, PDZ, and LIM domain protein 5, myopalladin and several other Z-disk proteins. In addition to their structural function, these proteins are known to play a role in signal transduction [47]. A number of signaling proteins including protein kinases and phosphatases are concentrated at the Z-disk where they interact with other Z-disk proteins which function as sensors of mechanical stress [47, 48]. In particular, PKC*ε* is localized to the Z-disk [49] and it translocate to the nucleus upon biomechanical stress [50, 51]. PKC*ε* has also been implicated in the cardioprotective effect of RIPC [29] and its activation is cardioprotective [52]. PDZ and LIM domain protein 5 (Table 1) has been shown to interact with PKC by scaffolding PKC to the Z-disk region [53]. Plakophilin 2 (PKP2) (Table 1) also serves as scaffold for PKC*α* signalling [54, 55].

Obscurin is a modular protein of ~800 kD which contains a GTPase nucleotide exchange factor (GEF) domain that provides a possible link between the sarcomere and the G-protein regulated pathways which control the formation of new myofibrils [56]. Obscurin also binds to ankyrin 1, which links the sarcoplasmic reticulum to the sarcomere and appears to be involved in the regulation of ryanodine receptor distribution [57]. Myozenin-2 is a calcineurin-binding protein and plays an important role in hypertrophic cardiomyopathy through its effect on calcineurin activity [58]. It has also been shown to protect against angiotensin-II induced cardiac hypertrophy [59].

What remains unclear is how myozenin-2 is regulated posttranscriptionally. A large scale phosphorylation analysis reported phosphorylation of myozenin-2 on T-107 and T-111 from mouse liver [60]. These modifications were also detected in the heart along with two other phosphorylation sites on S106 and S116 [61, 62]. In this study we detected increased level of phosphorylation of myozenin-2 on T-107, S-106, and S-101 immediately following RIPC compared to control (Table 3). Phosphorylation of myozenin-1 and myozenin-2 has been proposed to occur by protein kinase A (PKA) at residues within PDZ-binding motif [63]. It is known that PKA phosphorylates several sarcomeric proteins during stimulation of *α*-adrenergic receptors on myocytes [64]. Additionally activation of PKA could be responsible for the increased phosphorylation of ryanodine receptor 2 (Table 1) and troponin I (TnI) (Table 2). Phosphorylation of TnI by PKA is seen upon stimulation of the heart by *β*-agonists which results in a decrease in the Ca^2+^ sensitivity of muscle contraction [65]. It is worth noting that in one of the runs (series 2) we found decreased phosphorylation in cAMP dependent regulatory, type I, alpha unit of protein kinase (Table 2). Whether this would alter the activity of PKA is not presently known.

In conclusion, this study reports the novel finding that RIPC triggers changes in phosphorylation levels of cardiac proteins including several located to the Z-disk area of the sarcomere. Of particular interest is p-myozenin-2 which we found to be significantly higher in RIPC compared to control hearts. The increased phosphorylation was seen at different phosphorylation sites. Z-disk proteins are involved in signalling pathways and may protect the heart against R/I. Additionally; several kinases involved in survival signalling (e.g., PKA, PKC, and PKG) are also linked to the phosphorylation of these proteins. Further studies are needed to identify their role in cardioprotection during RIPC.

## Figures and Tables

**Table 1:** RIPC-induced changes in phosphoproteins measured using TMT. Data shown are for Series 1 (3 control and 3 RIPC). Fold change was calculated by taking average values for control and for RIPC.

Phospho-proteins	Protein accession	Fold change	*P* value	*P* value normalized to p-GAPDH
Myozenin-2	Q9JJW5	1.7	0.021*	0.043*
PDZ and LIM domain protein 5	E9Q8P5	1.3	0.003*	0.057
Protein Pkp2	E0CX59	1.3	0.008*	0.012*
Protein Tns1	E9Q0S6	1.3	0.006*	0.035*
Apoptosis-inducing factor short isoform 2	Q1L6K5	1.2	0.033*	0.040*
Isoform 2 of AP2-associated protein kinase 1	Q3UHJ0-2	1.4	0.014*	0.013*
Isoform 3 of synaptopodin	Q8CC35-3	1.3	0.036*	0.094
Ryanodine receptor 2	E9Q401	1.2	0.043*	0.196
Obscurin	F7DCJ0	1.2	0.012*	0.32
Alpha-T-catenin isoform X	A4GE65	1.2	0.033*	0.159
Ubiquitin carboxyl-terminal hydrolase	B1AY13	1.2	0.029*	0.031*
Sorbin and SH3 domain-containing protein 2	B2RXQ9	1.4	0.007*	0.050
ENH isoform 1e	D9J302	1.2	0.003**	0.051
p53-induced protein with a death domain	Q9ERV7	1.3	0.034*	0.092
Calnexin	P35564	1.2	0.071	0.013*

*P*-value calculated using unpaired *t*-test (*P < 0.05 considered as significant). *P* values between 0.05–0.1 indicate strong trend.

**Table 2:** RIPC-induced changes in phosphoproteins measured using TMT. Data shown are for the second group of hearts (Series 2; 3 control and 3 RIPC). Fold change was calculated by taking average values for control and for RIPC.

Phosphoproteins	Protein accession	Fold change	*P* value	*P* value normalised to p-GAPDH
Myozenin-2	Q9JJW5	1.9	0.015*	0.020*
Tropomyosin alpha-1 chain	P09493	1.5	0.036*	0.08
Troponin I	P48787	1.6	0.029*	0.043*
M-protein	O55124	1.4	0.023*	0.016*
Eif4g1 protein	Q8R2V4	1.3	0.010*	0.040*
Isoform 2 of titin	A2ASS6-2	1.6	0.061	0.12
Junctophilin-2	Q9BR39	1.7	0.070	0.053
Isoform A3B of troponin T, cardiac muscle	P50752-2	1.2	0.06	0.064
Ataxin-2	F6V8M6	0.8	0.005*	0.043*
Isoform Tau-C of microtubule-associated protein tau	P10637-4	0.8	0.008*	0.050
Centrosomal protein of 170 kDa	H7BX26	0.8	0.031*	0.072
Protein kinase, cAMP dependent regulatory, type I, alpha	A2AI69	0.7	0.019*	0.031*
THUMP domain-containing protein 1	Q99J36	0.8	0.028*	0.09
CDKN2A-interacting protein	Q9NXV6	0.8	0.060	0.103

*P*-value calculated using unpaired *t*-test (*P < 0.05 considered as significant). *P* values between 0.05–0.1 indicate strong trend.

**Table 3:** RIPC-induced changes in phosphorylation of myozenin-2 (protein accession: Q9JJW5) as per phosphorylation sites (amino acids shown in small bold and underlined letters). Phosphorylated myozenin-2 was measured using TMT isobaric mass tagging. Data shown are for series 1 (3 control and 3 RIPC). Fold change was calculated by taking average values for control and for RIPC.

Amino acid sequence of fragments	Modified residue(s)	Fold change	*P* value	*P* value normalised to p-GAPDH
vDGSNLEGGSQQGPS**t**PPNTPDPR	T107	1.67	0.021*	0.043*
vDGSNLEGGSQQGP**s**TPPNTPDPR	S106	1.55	0.007*	0.025*
vDGSNLEGG**s**QQGPSTPPNTPDPR	S101	1.34	0.017*	0.078
vDGSNLEGG**s**QQGP**s**TPPNTPDPR	S101/S106	1.15	0.290	0.169
vDGSNLEGG**s**QQGPS**t**PPNTPDPR	S101/T107	1.09	0.665	0.637
vDGSNLEGGSQQGP**s**TPPN**t**PDPR	S106/T111	1.15	0.290	0.169
vDGSNLEGGSQQGPS**t**PPN**t**PDPR	T107/T111	1.09	0.665	0.637

*P* value calculated using unpaired *t*-test (*P < 0.05 considered as significant).
